# Association analysis between polygenic risk scores and traits: practical guidelines and tutorial with an illustrative data set of schizophrenia

**DOI:** 10.3389/fpsyt.2025.1621972

**Published:** 2025-08-21

**Authors:** Itziar Irigoien, Patricia Mas-Bermejo, Sergi Papiol, Neus Barrantes-Vidal, Araceli Rosa, Concepción Arenas

**Affiliations:** ^1^ Department of Computation and Artificial Intelligence, Euskal Herriko Unibertsitatea (UPV/EHU), Donostia, Spain; ^2^ Zoology and Biological Anthropology Section of the Evolutionary Biology, Ecology and Environmental Sciences Department, Universitat de Barcelona (UB), Barcelona, Spain; ^3^ Institut de Biomedicina de la UB (IBUB), Barcelona, Spain; ^4^ Centre for Biomedical Network on Mental Health (CIBERSAM), Instituto Salud Carlos III, Barcelona, Spain; ^5^ Institute of Psychiatric Phenomics and Genomics (IPPG), University Hospital, Ludwig-Maximilians-Universität München (LMU) Munich, Munich, Germany; ^6^ Max Planck Institute of Psychiatry, Munich, Germany; ^7^ Department of Clinical and Health Psychology, Universitat Autònoma de Barcelona, Barcelona, Spain; ^8^ Sant Pere Claver- Fundació Sanitària, Barcelona, Spain; ^9^ Statistics Section of the Department of Genetics, Microbiology and Statistics, Universitat de Barcelona (UB), Barcelona, Spain

**Keywords:** polygenic risk score, statistical analysis, covariates, schizophrenia, psychotic-like experiences

## Abstract

Most methodological Polygenic Risk Score (PRS)-related papers explain the laborious process of computing the PRS in great depth. Afterwards, as a last step, it is generally described that to test a possible association between a PRS and a trait of interest, an analysis through regression models (linear or logistic, depending on data type) should be carried out adjusting for covariates (e.g., sex, age, clinical information, or genetic ancestry-based Principal Components). When covariates are included, measurements such as the increment on the variance explained by the addition of the PRS to the model or the significance of the PRS term are usually reported. However, the association study between PRSs and a trait is a complex concern that requires proper modeling and analysis, since interactions and validation conditions represent crucial aspects. Even though excellent papers explain how to use and interpret the results obtained with such regression models, sometimes important information from the previously calculated PRS may be lost, partly due to the automation of analyses. With this guide, we intend to fill a gap in association studies between PRSs and a trait and to facilitate the analysis, obtaining statistically correct results. It contains a motivating real data case analyzed exhaustively to illustrate how to face a real analysis. Besides, it is accompanied by four examples, called *Working Examples*, which present different situations the researcher may encounter along with the R code for analyzing all these data sets and the corresponding application of the steps in this guide.

## 1 Introduction

A Polygenic Risk Score (PRS) is an estimated value of an individual’s genetic susceptibility to a trait, condition, or disease, and it is calculated based on the results of a Genome-Wide Association Study (GWAS). Once it is calculated, a typical analysis includes testing for association between the PRS and a trait through a linear or logistic regression model. In the most common situation, the models include covariates such as sex, age, clinical diagnosis, or genetic ancestry-based Principal Components used to control for potential population substructure, among others. Then, to evaluate the effect of the PRS alone, two models are usually considered: the so-called *null or baseline* model and the *full* model. The *null* model consists of the trait as the response variable and the covariates as predictor variables. The *full* model incorporates the PRS into the *null* model as a predictor. They are nested models, usually with only one different term, the PRS. Then, both models are analyzed, and the significance of the PRS and the increase in the explained variance between the models are evaluated. Usually, the value of the coefficient of determination *R* ^2^, or the adjusted coefficient of determination, $R_{adj}^{2}$ , is reported in the case of a continuous trait. Likewise, if it is a binary trait, the value of a *pseudo*-*R* ^2^ coefficient of Nagelkerke is reported.

There are different approaches to computing a PRS. The traditional Clumping + Thresholding (C+T) method enables the selection of independent variants through LD-pruning to avoid redundancy and the inclusion of more or less significantly associated variants with the discovery trait by establishing different GWAS *p*-value thresholds, often resulting in the generation of several PRSs according to the thresholds used (1). However, more advanced methods have been developed recently that re-weight the SNP effect sizes from the GWAS summary statistics, applying some form of shrinkage and usually allowing for obtaining a single PRS (2).

Although the literature includes excellent papers explaining how to use and interpret regression models (3–9), a guideline for conducting PRS association studies is necessary to assist non-statistician researchers in performing the statistical analyses correctly. Below are the guidelines for conducting these association analyses after calculating the PRS. Therefore, this guide does not focus on the calculation process used to obtain a PRS but rather on analyzing the association between a trait and a previously calculated PRS. This guide features a detailed real case to illustrate how to approach a real analysis. Additionally, it is accompanied by four examples, called *Working Examples*, which gradually present all the steps explained in increasing order of difficulty. Moreover, the data sets, R code for the analysis, and PDF files containing the results with software output are available at: https://github.com/ItziarI/SupportingMaterial-for-the-guide.

**Figure 1:**
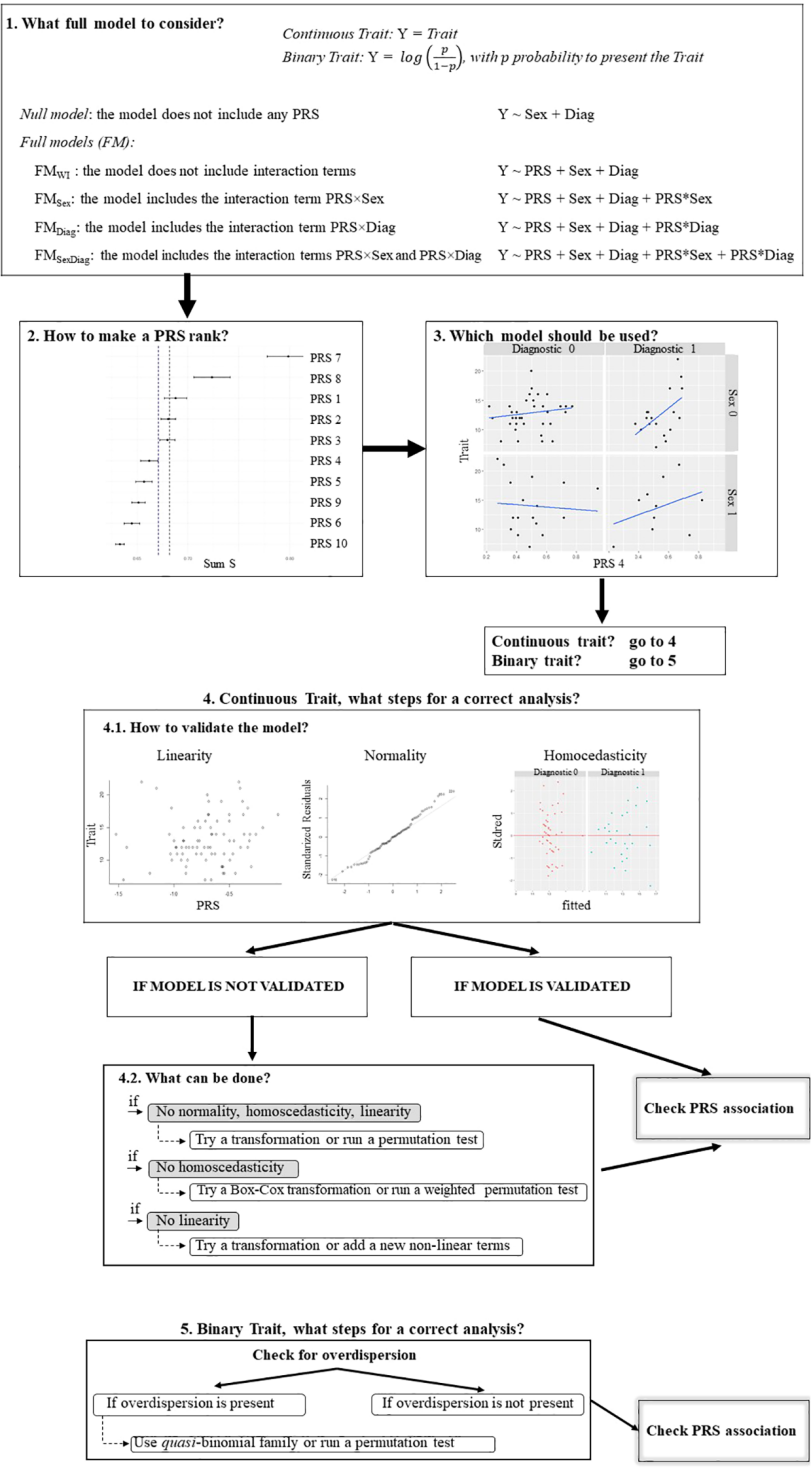
Workflow of the association analysis described in Section 2. Each panel is associated with one of the steps. Panel 1: describes the different possible models to consider, as explained in subsection 2.1; Panel 2: shows a plot related to subsection 2.2 where a semi-automated procedure to reduce the number of PRSs to be analyzed is presented; Panel 3: related to subsection 2.3 where it is discused how stablish the most appropiate model among all the possible ones; Panel 4: in this panel subsection 2.4 is summarized with the validation of the model and the different strategies to follow depending if the model is validated or not; Panel 5: shows the steps that should be followed for a correct analysis with a binary trait, as it is described in subsection 2.5.

The definitions needed to follow the next sections are reported in **Box 1**. The different steps of the proposed guide are presented in the next section and summarized in **Figure 1**.

Box 1 **Definitions.**

*Null model:* the trait is the response, and the covariates are the predictors.

*Full model:* the trait is the response; the PRS and covariates are the predictors.

*Nested model: a* regression model that includes only a subset of the predictor variables from the other regression model.

*Coefficient of Determination R^2^*: it gives the percentage variation in the response variable explained by the predictor variables. The range is 0 to 1 (i.e., 0% to 100% of the variation in the response can be explained by the predictor variables).

*Adjusted Coefficient of Determination* $R_{adj}^{2}$ : it also indicates the goodness of the model, but adjusts for the number of predictors.

*Pseudo-R^2^ coefficient of Nagelkerke:* similar to *R^2^* when the response variable is binary.

*Akaike Information Criterion (AIC):* metric used to compare the fit of different regression models. The model with the lowest AIC offers the best fit.

*Discrimination coefficient D:* measure of the discriminant capacity of the two-class logistic regression model. The range is between 0 and 1, with large values indicating the logistic model discriminates better between the classes.

*Logit values:* also known as “log-odds”, is the natural logarithm of the odds concerning one event. If *p* is the probability of an event, the odds is given by the ratio *p/(1-p).* The bigger this value, the greater the chances for the event to occur.

Validation of a linear regression model:

- *Linearity:* there is a linear relationship between each predictor and the response.
- *Normality:* the errors follow a normal distribution with a mean equal to zero
  ○ *QQ-plot:* short for “quantile-quantile” plot, is used to assess whether or not a set of data potentially came from some theoretical distribution.
  ○ *Shapiro’s test:* is used to determine whether or not a given dataset follows a normal distribution (H_0_: data is normally distributed *vs* H_1_: data is not normally distributed)

- Constant variance or homoscedasticity: constant variance for all subjects
  ○ *Levene’s test:* is used to determine whether or not the variance is constant
  ○ *Heteroscedasticity:* there is no homoscedasticity

*Skewed data:* if one tail is longer than the other, the distribution is skewed (or asymmetrical)

*Nonparametric approach:* a method that makes statistical inference without regard to any underlying distribution

*Permutation test:* permutation tests work by resampling the observed data many times to determine a p-value for the test

*Box-Cox transformation:* it is a useful family of transformations to convert a non-normal behaving data set into an approximately normal distribution.

*Overdispersion:* occurs when the discrepancies between the observed responses and their predictions according to the model are larger than what the binomial model would predict.

*Binomial and quasi-binomial distribution:* probability distributions that arise when counting the number of times an event of interest happens given a fixed number of trials. In the binomial distribution, the variance is completely determined by the probability of the event of interest. The quasi-binomial distribution has an extra parameter allowing additional variance compared to the binomial distribution.

## 2 Guidelines for the association analysis

### 2.1 Which *full* model should be considered?

The researcher should be aware of the possibility of different *full* models. In certain situations, it is necessary to consider models that incorporate interaction terms; however, only those interactions with biomedical meaning or interest for the researcher should be considered. Therefore, the number of models with interactions will not be large, as generally only those related to covariates such as sex, diagnosis, or age are considered. Therefore, for each PRS we can assume different *full* models. For instance, if there are two categorical covariates of interest, such as sex and diagnosis, four different *full* models (FM) can be considered (first panel in **Figure 1**):

- FM_WI_: the model without interaction terms.
- FM_Sex_: the model with the interaction term PRS×Sex.
- FM_Diag_: the model with the interaction term PRS×Diag.
- FM_Sex/Diag_: the model including the interaction terms PRS×Sex and PRS*×*Diag.

### 2.2 When and how to make a PRS ranking?

This section should only be considered if the researcher has used a PRS calculation method that has generated several PRSs to analyze (e.g., using several p-value thresholds in the C+T approach) and wants to determine which of these PRSs are of greatest interest before performing the association analysis to avoid having to analyze all of them. Instead, if the method generates only a single PRS, this step should be skipped, and the next step is in subsection 2.3.

We propose a semi-automated procedure to reduce the number of PRSs that need detailed analysis. If the trait is continuous, for each PRS and each possible *full* model (including and excluding interaction terms), calculate the coefficient of determination *R* ^2^ and let *S* be the sum of all of them. Similarly, if the trait is binary, calculate the *pseudo*-*R* ^2^ coefficient of Nagelkerke or the discrimination coefficient *D* for each *full* model and the sum *S* of these values. Next, rank the PRSs by *S* in decreasing order. This ranking is generated automatically, and the top PRSs, which explain more phenotypic variance across all *full* models, deserve a more careful analysis. Note that this sum incorporates information from all considered models and its use prevents the need to rank all possible models across different PRSs.

The PRSs should be analyzed individually, following the ranking established by *S* until no association is found for a given PRS. The possible gap in the *S* values between two consecutive PRSs can also be used as a stopping rule.

A visualization of this PRS rank is possible, as shown in panel 2 of **Figure 1**. The *x*-axis represents the *S* values, and vertical lines can be drawn at different points, such as the mean, median, or percentiles of *S*. Each horizontal line represents a PRS. Black dots symbolize the *S* value for each PRS, and adjacent whiskers represent the standard deviation of the *R* ^2^ values (*pseudo*-*R* ^2^ coefficient of Nagelkerke or the discrimination coefficient *D*, respectively) obtained from all possible *full* models. Thus, the length of these horizontal bars can be interpreted as an indicator of the importance of considering interactions between a PRS and the covariates.

### 2.3 Which model among all the possible ones is the most appropriate?

For a fixed PRS, it is necessary to determine which is the most appropriate *full* model. First, it must be determined if the model should contain interaction terms, and if so, which ones.

For a continuous trait, scatterplots of the trait and PRS by categorical predictors are useful for visually checking the homogeneity of slopes and determining whether interaction terms should be included. For example, panel 3 of **Figure 1** contains four scatterplots, each showing the relationship between a trait and PRS.4, separated by sex and diagnosis. Different slopes are observed. For diagnosis 1, the slopes increase (for both sexes 0 and 1); whereas for diagnosis 0, the slopes are very gentle, either slightly increasing (sex = 0) or slightly decreasing (sex = 1). Therefore, the interaction term “PRS×Diagnosis” should be included in the full model.

For a binary trait, compute the model with all possible interactions and plot the predicted logit values against the PRS. If interaction terms are relevant, different logit behaviors should appear.

At this point, a full model candidate is established; it is crucial to verify whether it meets the conditions required by the statistical analysis, as explained below. However, this validation is not typically performed in automated analyses.

### 2.4 For a continuous trait, what steps should be followed for a correct analysis?

If the outcome variable is a continuous trait, the focus must be placed on different issues (panel 4 in **Figure 1**).

#### 2.4.1 How is the candidate model validated?

To validate the *full* model for a continuous trait, linearity (by scatter plots of trait and PRS), normality (by QQ-plots and Shapiro’s test), and constant variance or homoscedasticity (by Levene’s test) must be checked. The fourth validity condition, the independence of the observations, is generally guaranteed by the design of the experiment itself. Remember that the analysis does not assume normality for either the predictors or the trait. The assumption is that errors are normally distributed, with a mean of zero. The verification of these conditions is relevant in the context of inference regarding the interpretability and significance of the coefficients. For instance, heteroscedasticity tends to produce smaller *p*-values than they should be (10). Consequently, this problem can lead to the conclusion that a model term is statistically significant when it is not. Furthermore, heteroscedasticity persists as a problem, regardless of sample size.

#### 2.4.2 What can be done if any validation condition fails?

Different strategies will be considered depending on the failed validation condition (panel 4.2 in **Figure 1**).

When it is suspected that the normality of errors fails, but the model is homoscedastic, and the linearity is maintained, try transforming the response variable. For instance, take the logarithm for positively skewed data or the square root for more moderate skewness situations. Such a transformation aims to rebuild a valid candidate model that allows for assessing the association between the PRS and the trait. However, a nonparametric approach is also possible. A permutation test allows researchers to measure whether the increase in the determination coefficient observed between the *null* model and the candidate *full* model is significant. However, with this type of nonparametric approach, it will not be possible to establish if the association is significant or how the variation (of one unit) of the PRS affects the trait. Furthermore, it will not be possible to evaluate the different behavior of the PRS in the groups generated by the categorical variable.

If heteroscedasticity is suspected but the linearity of the model is maintained, it would be advisable to try a Box-Cox transformation of the response variable. The Box-Cox transformations for different values of *λ* are given by: (trait – 1)/*λ*, if *λ* ≠ 0, and log(*trait*) if *λ* = 0. Again, it is possible to consider a nonparametric approach. Now, use a weighted permutation test to measure whether the increase in the determination coefficient observed between the *null* and the selected *full* model is significant. During its construction process, the weighted permutation test considers heteroscedasticity, making the underlying residuals interchangeable.

If the problem lies in the linearity, try a transformation of the response variable or include new non-linear terms.

#### 2.4.3 How is a possible association established?

Once the PRS, the model, and its validation have been established, analyze the possible association by checking the value and significance of the regression coefficients. At this point, great care must be taken when interpreting the lists provided by the most common software. This is extremely important if the model contains interaction terms, as the value of the PRS coefficient and its significance can vary depending on the group to which each individual belongs.

The detailed *Working Examples* 1-3, included in the Supporting material (see Section 8), aim to understand the necessity of performing these steps and how to perform them correctly (*Working Example* 1: Continuous trait, and model fulfilling all assumptions; *Working Example* 2: Continuous trait and steps taken to address the non-normality of errors; *Working Example* 3: Continuous trait and the steps taken to address issues in the initial fitted model with non-constant variance.)

### 2.5 For a binary trait, what steps should be followed for a correct analysis?

If the outcome variable is a binary trait, given a PRS, check overdispersion once the full model has been established. When the ratio comparing the residual deviance with the degrees of freedom is considerably larger than 1, the assumption of binomial variation is violated, and then overdispersion occurs. Overdispersion can also be checked by fitting a logistic regression under two different models using a binomial and a *quasi*-binomial distribution, respectively. If there is statistical evidence that the expected variance of the two models is significantly different, we can conclude that there is overdispersion. If the candidate *full* model has no overdispersion, check the significance of the PRS, and analyze the possible association with the trait.

On the contrary, if overdispersion is detected (panel 5 in **Figure 1**), a simple solution to overdispersion would be to estimate an additional parameter that indicates the amount of overdispersion and specify a quasi-binomial family instead of a binomial in the logistic regression model. Again, when the model includes interaction terms, it is crucial to interpret correctly possible significant associations (see the detailed explanations in *Working Example* 4).

When working with binary traits, it is also possible to use a nonparametric approach by a permutation test to assess whether the increase in the *pseudo*-*R* ^2^ coefficient of Nagelkerke or the coefficient of discrimination *D* is significant. However, the limitations of this approach must be kept in mind, as it will not be possible to determine regression coefficients or their significance.

### 2.6 Is it necessary to consider all the steps mentioned above?

The answer is Yes. Not considering interaction terms may mask associations between PRS and trait in some groups determined by the corresponding categorical covariate. Ignoring model validation can lead to negative consequences, such as mistakenly concluding that the PRS is significantly associated with the trait when it isn’t. The *Working Examples* and the real data set demonstrate how poor analysis leads to erroneous conclusions. Finally, the real data set illustrates the difficulties that can arise with real data and how they can be solved following this guide, for both continuous and binary traits.

## 3 Analysing a real data set

The real data set contains a PRS for psychotic-like experiences (PLEs) computed for 227 healthy individuals, including 64 men (28.1%) and 164 women (71.9%). PLEs are similar to psychotic experiences to those experienced by patients with schizophrenia, but are found in an attenuated form in healthy subjects. PLEs are considered to be normally distributed in the general population, with just a few individuals presenting high levels of PLEs and thus being the ones at risk of developing psychosis (11–13). The PRS in this example was calculated based on the latest GWAS on PLEs (14) with the classical Clumping + Thresholding method (1), using 106 *p*-value thresholds ranging from 5×10^−8^ to 1 to allow us to exemplify the second step of this guide. This motivational example aims to determine which PRSs for PLEs (PLE-PRSs) are associated with a phenotypic measure of PLEs in non-clinical individuals.

Specifically, participants in this data set completed the Community Assessment of Psychic Experiences (CAPE) questionnaire (15), which assesses three dimensions of PLEs: positive, negative, and depressive dimensions. For this tutorial, we used the information on the positive and negative dimensions of PLEs (CAPE Positive and CAPE Negative, respectively).

We considered CAPE Negative as a continuous trait and CAPE Positive as a binary trait separating individuals with high and low levels of PLEs to illustrate how to apply the steps of this guide for both linear and logistic regression models, respectively. In both situations, sex, age, and the first two ancestry-based Principal Components are used as covariates. A larger number of PCs have not been included as usual since it would not add meaningful insight for our purpose and would only lengthen the results tables. The descriptive characteristics of this real data set are in **Table 1**.

**Table 1:** Descriptive characteristics of the real data set.

Variables	Mean	SD	Observed range	Possible range
Age	19.95	2.801	17 - 44	
CAPE Positive	8.48	5.042	0 - 23	0 - 60
CAPE Negative	10.31	5.619	0 - 35	0 - 42
CAPE Depressive	5.94	2.957	1 -18	0 - 24

The question is: out of the 106 PRSs that were built, which are the most important to carry out a detailed association analysis? Furthermore, does it make sense to consider models that include interaction terms, for example, between PRS and sex or between PRS and age? Note that if these interaction terms are included and they are significant, we will obtain information regarding how the increase/decrease in PRS values affects the Positive CAPE or Negative CAPE values depending on whether the individual is male (coded by 0) or female (coded by 1), or depending on their age. This information will be lost if interactions are not introduced into the model.

For a better understanding of the results detailed below, it is recommended to run the scripts (see Section 8) simultaneously.

### 3.1 CAPE negative as the trait

Considering the biomedical meaning of the analysis, only the *full* models FM_WI_ (without interaction terms) and FM_Sex_ (including the interaction term PRS×Sex) are considered. For each model, we calculated the coefficient of determination *R^2^* and the sum $S\;=\;R_{WI}^{2}\;+\;R_{Sex}^{2}$ . We ranked the PRSs, and we identified PRS.13 (i.e., the PRS derived from GWAS *p*-value threshold = 0.07) as the top-ranked. Note that if the method used to compute the PLE-PRSs had been one that generates a single PRS, this step would have been omitted.

**Figure 2:**
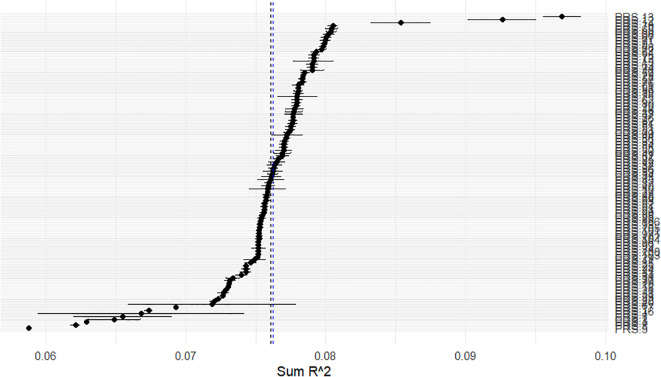
Real data set. On the x-axis, the *S* values described in section 2.2 are plotted. On the y-axis, the PRSs are ranked in decreasing order according to *S*. Black dots represent the *S* for each PRS, and the adjacent whiskers represent the standard deviation of the R^2^ values obtained for the possible *full* models (including and not including interaction terms).

**Figure 3:**
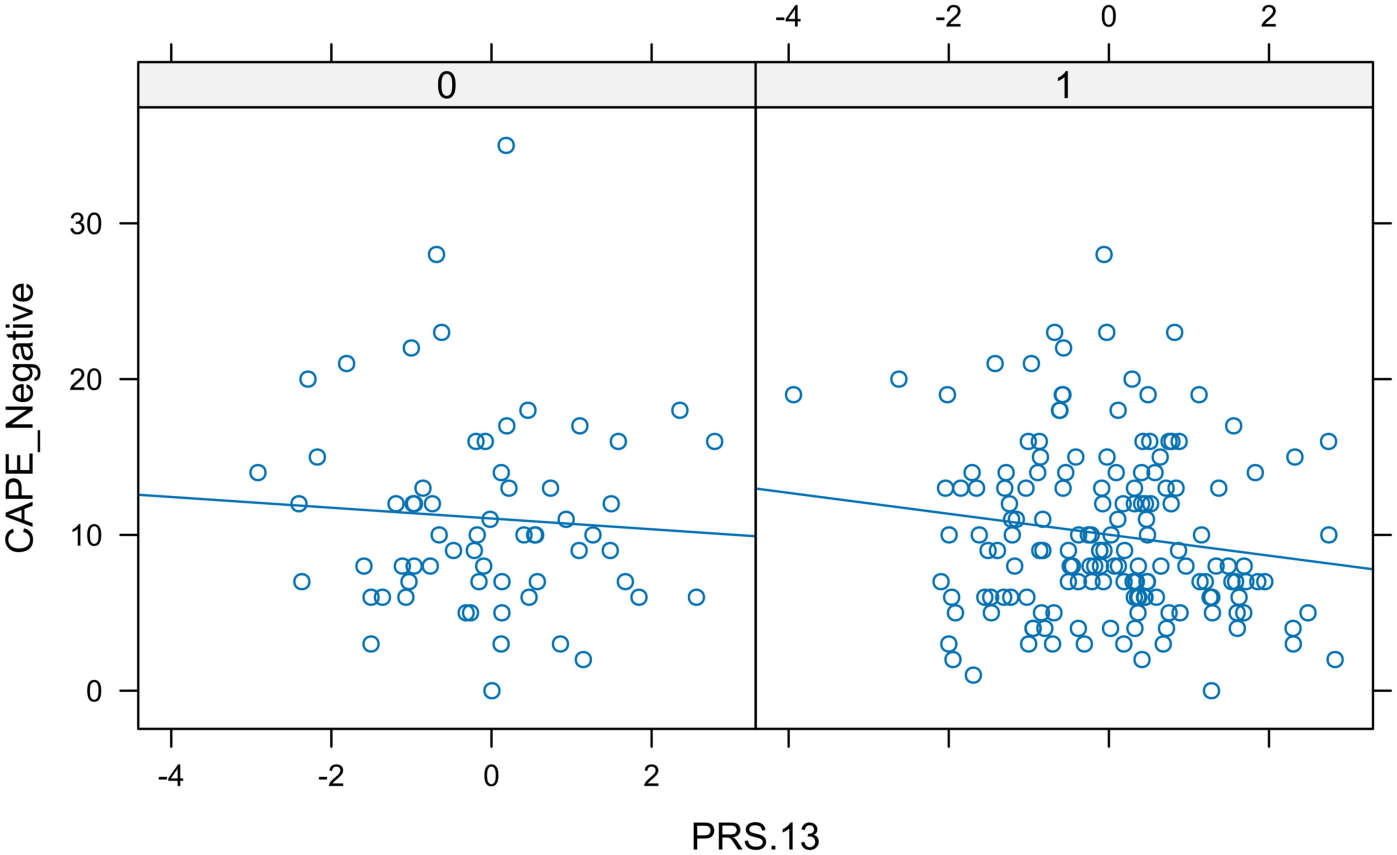
Real data set. In each panel, a scatter plot showing the relationship between negative CAPE and PRS.13 separated by sex. The similar slope of the lines indicated the lack of interaction.

For PRS.13, the first of the PRSs rank, the short length of the horizontal bars in **Figure 2**, and the scatter plots of Trait *vs.* PRS.13 according to sex (**Figure 3**), which was the only categorical predictor, indicated the lack of interaction. Thus, we considered the following model, FM_WI_: CAPE_Neg *versus* PRS.13 + Sex + Age + PC1 + PC2, as a candidate full model.

**Figure 4:**
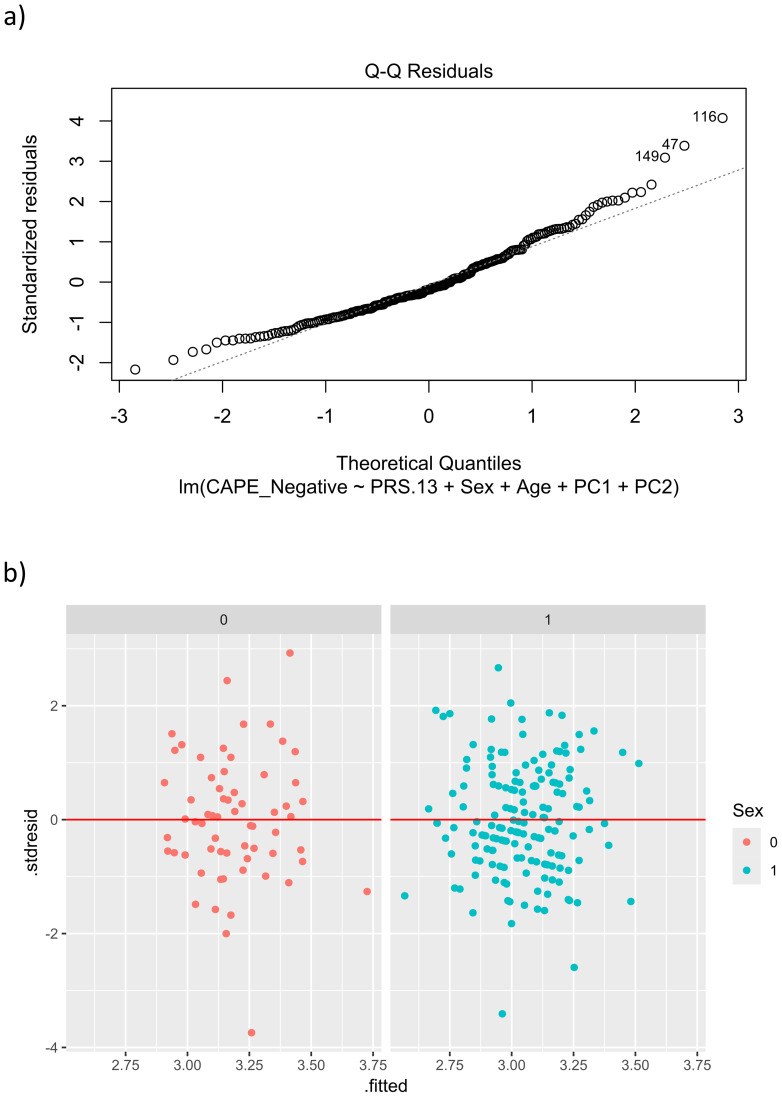
Real data set. Validation conditions for the model FM_WI_: CAPE_Neg *versus* PRS.13 + Sex + Age + PC1 + PC2. **(a)** QQ-plot for normality, deviations from the diagonal line indicate that the errors’ distribution differs from a normal distribution. **(b)** Plot for homocedasticity after squared root transformation, each panel shows the scatter plot of residuals versus fitted values according to sex.


The residuals did not follow a normal distribution (Shapiro test *p*-value = 4.335e-06; see **Figure 4a**), so a square root transformation was applied. With this transformation, the normality condition is already met (Shapiro test *p*-value = 0.0811), and the homoscedasticity assumption also holds (Levene’s test *p*-value = 0.5846; **Figure 4b**). The results showed that PRS.13 was significantly (*p*-value = 0.045) related to the *sqrt*(CAPE_Neg) in the following way:

$$\begin{array}{c} \hat{\sqrt{\text{CAPE}\_\text{Neg}}}=3.013-0.101\cdot PRS.13-0.142\cdot Sex+0.009\\ \cdot Age+3.797\cdot PC1+8.409\cdot PC2 \end{array}$$

and then,


$$\begin{array}{c} \hat{\text{CAPE}\_\text{Neg}}=(3.013-0.101\cdot PRS.13-0.142\\ \cdot \text{S}ex+0.009\cdot Age+3.797\cdot PC1+8.409\cdot PC2{)}^{2} \end{array}$$

The effect of the change of one unit in PRS.13 in CAPE_Neg can be measured as follows. Compute the estimations at the indicated values of PRS.13


$$\hat{(\text{CAPE}\_{\text{Neg}}_{0}})\;\text{and PRS }.13+1\hat{(\text{CAPE}\_{\text{Neg}}_{1}}):$$

$$\begin{array}{c} \hat{\text{CAPE}\_{\text{Neg}}_{0}}=(3.013-0.101\cdot PRS.13-0.142\\ \cdot \text{S}ex+0.009\cdot Age+3.797\cdot PC1+8.409\cdot PC2{)}^{2} \end{array}$$

and


$$\begin{array}{c} \hat{{\text{CAPE}}_{{\text{Neg}}_{1}}}=(3.013-0.101\cdot \left(PRS.13+1\right)-0.142\cdot \text{S}ex+0.009\\ \cdot Age+3.797\cdot PC1+8.409\cdot PC2{)}^{2}={\left(-0.101+\sqrt{\hat{\text{CAPE}\_{\text{Neg}}_{0}}}\right)}^{2} \end{array}$$

then, difference


$$\hat{\text{CAPE}\_{\text{Neg}}_{1}}-\hat{\text{CAPE}\_{\text{Neg}}_{0}}$$

is given by:


$${\left(-0.101\right)}^{2}+2\cdot \left(-0.101\right)\cdot \sqrt{\hat{\text{CAPE}\_{\text{Neg}}_{0}}}$$

Thus, if the current CAPE_Neg is, for instance, 5, 10, or 25, increasing one unit in PRS.13 is associated with a change of −0.4415, −0.6286, and−0.9998 in CAPE_Neg, respectively.

With the permutation test, we obtained a significant (*p*-value = 0.0033) increase of 0.0197 in the coefficient of determination when the PRS.13 was included in the baseline model.

**Figure 5:**
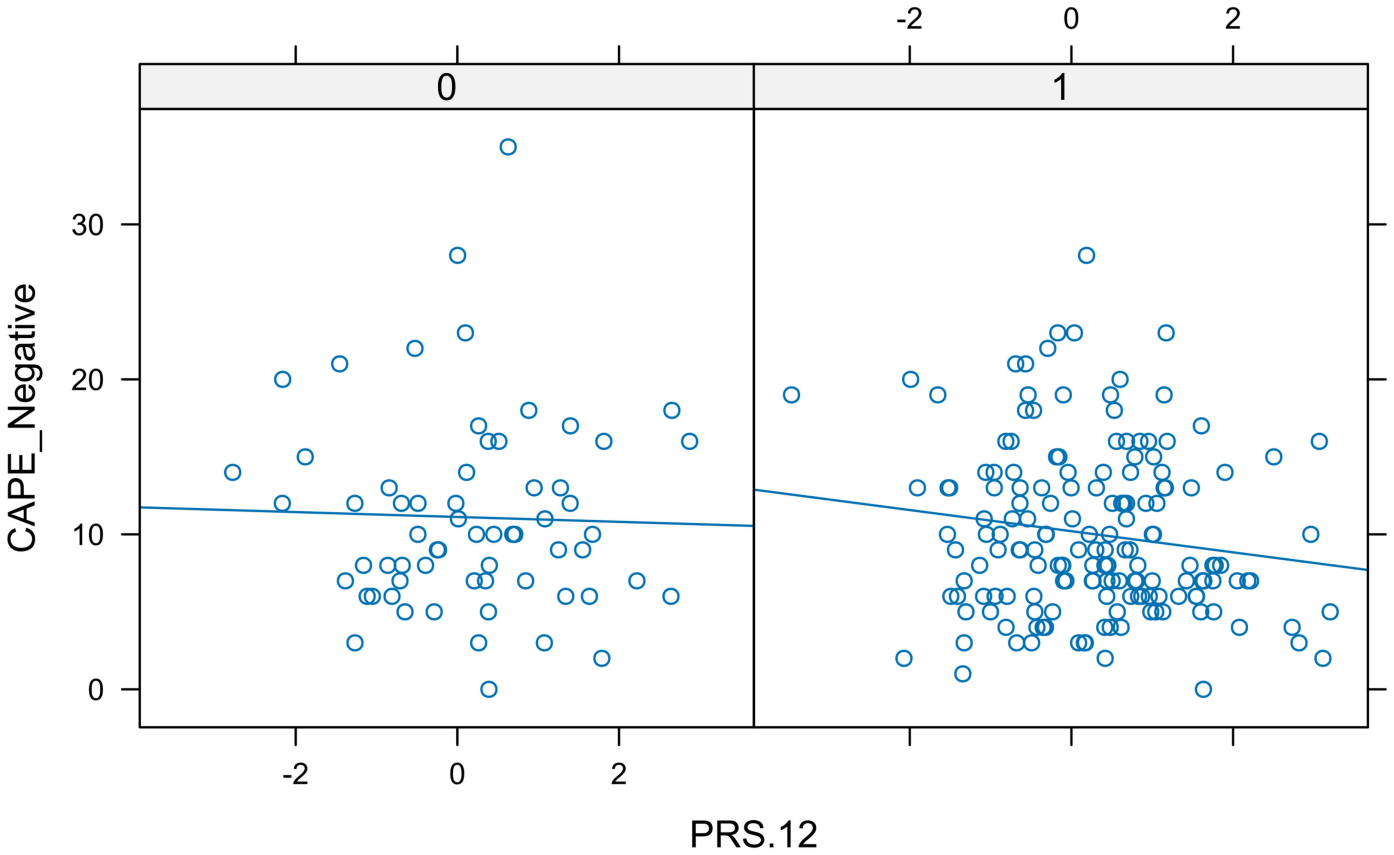
Real data set. In each panel, a scatter plot shows the relationship between CAPE negative and PRS.12 separated by sex. The different slopes of the lines indicated the presence of interaction.

The next PRS in the ordered list was PRS.12, and **Figure 5** indicated that the interaction term should be considered. Again, the residuals did not follow a normal distribution, and neither with a transformation nor using the permutation test, a significant association was found between PRS.12 and CAPE_Neg (*p*-value = 0.7170 and 0.0956, respectively). Therefore, there is no need to study more candidate PRSs.

### 3.2 CAPE positive as the trait

**Figure 6:**
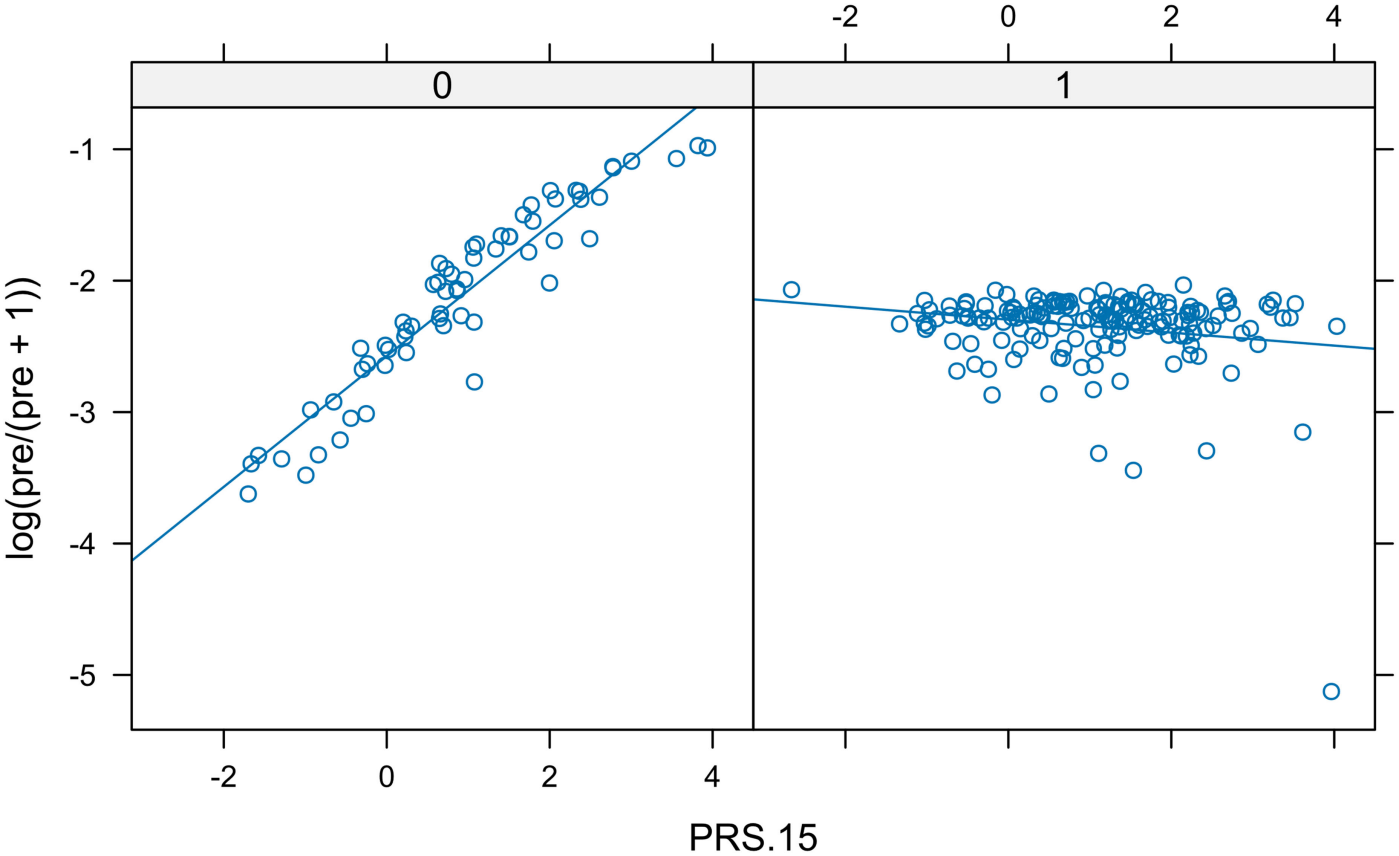
Real data set. In each panel, a scatter plot of fitted logit predictions shows the relationship between positive CAPE and PRS.15 separated by sex. The different slopes of the lines indicated the presence of interaction.

Finally, we considered CAPE Positive as a binary trait separating individuals with high and low levels of PLEs. This scale has no fixed criterion to decide who scores high and who scores low. However, based on its histogram, we considered a threshold of 15 to indicate those individuals who would score high (the 1% of the sample). According to the discrimination coefficient *D*, the top PRS was PRS.15 (i.e., the PRS derived from GWAS *p*-value threshold = 0.09). The plot of the predicted logit values against the PRS.15 indicated that the interaction term was relevant (**Figure 6**).

As overdispersion was not detected (*p*-value = 0.2651), we analyzed the possible association with CAPE_Pos. Note that in **Table 2** (standard output given by the R package), values associated with PRS.15 for Sex=0 are in the second line (coefficient b_1_). Those for Sex=1 are, for the intercept, in lines 1 and 3 (coefficients b_0_ and b_3_); for the PRS.15 coefficient in lines 2 and 7 (coefficients b_2_ and b_6_). Thus, the PRS.15 coefficient that varies depending on the sex (**Table 2**), is given by:

**Table 2:** For the real data set with a binary Trait, logistic regression results for CAPE Positive *versus* PRS.15, considering interaction with Sex.

Model terms	Parameter	Null hypothesis	Estimate	Std. error	z value	*p*-value
Intercept	b_0_	b_0_ = 0	0.0549	2.2242	0.025	0.9803
PRS.15	b_1_	b_1_ = 0	0.7463	0.2900	2.574	0.0101
Sex1	b_2_	b_2_ = 0	0.1390	0.6671	0.208	0.8350
Age	b_3_	b_3_ = 0	-0.1183	0.1070	-1.106	0.2686
PC1	b_4_	b_4_ = 0	1.9513	14.5402	0.134	0.8932
PC2	b_5_	b_5_ = 0	2.8671	14.9406	0.192	0.8478
PRS.15:Sex1	b_6_	b_6_ = 0	-0.7487	0.3588	-2.087	0.0369

- If Sex = 0,

$$\begin{array}{c} \hat{\text{log}\left(\frac{p}{1-p}\right)}=0.055+0.746\cdot \text{PRS}.15-0.118\\ \cdot \text{Age}+1.951\cdot \text{PC}1+2.867\cdot \text{PC}2 \end{array}$$

- If Sex = 1,

$$\begin{array}{c} \hat{\text{log}\left(\frac{p}{1-p}\right)}=\left(0.055\;+\;0.139\right)+\left(0.746-0.749\right)\cdot \text{PRS}.15-0.118\\ \cdot \text{Age}+1.951\cdot \text{PC}1+2.867\cdot \text{PC}2 \end{array}$$

That means that for those with Sex=1, the PRS.15 is not related (*p*-value = 0.9910) to CAPE_Pos with odds = exp( − 0.002) = 0.998. For those with Sex = 0, the model indicates that PRS.15 is significantly associated (*p*-value = 0.0101) with the CAPE_Pos with a coefficient 0.7463, so the odds increase exp(0.7463) = 2.109 for an incremental of one unit in PRS.15 (see **Table 3**). It is very important to note that if the interaction is not included in the model, meaning that if the model assumes the association of PRS.15 to be the same for both sex categories, then the association of PRS.15 with CAPE_Pos might be lost (*p*-value = 0.0764). Most importantly, the different behavior regarding sex would not have been detected.

**Table 3:** For the real data set with a binary Trait, and according to sex, parameters, null hypothesis, estimates, standard errors, z statistics, and *p*-values using model FM_Sex_.

Sex condition	Parameter	Null hypothesis	Estimate	Std. error	z value	*p*-value
Sex 0	b_1_	b_1_ = 0	0.7463	0.290	2.574	0.0101
Sex 1	b_1_ + b_6_	b_1_ + b_6_ = 0	-0.0024	0.212	-0.011	0.9910

The analysis continued, studying the possible association with the following PRS from the list obtained. For the first six ranked PRSs, **Table 4** shows a clear association with CAPE_Pos in group Sex equal to 0, but not when Sex is 1. Note that all these associations would have been lost if the interaction term had not been considered.

**Table 4:** For the real data set with a binary Trait and according to sex, estimates, *p*-values, and odds using model FM_Sex

PRS	Sex	Estimate	*p*-value	*p*-adjusted	Odds
PRS.15	0	0.746	**0.0101**	**0.0367**	2.109
	1	-0.002	0.9910	0.9910	0.998
PRS.16	0	0.719	**0.0127**	**0.0367**	2.052
	1	0.058	0.7810	0.9112	0.060
PRS.17	0	0.657	**0.0204**	**0.0476**	1.921
	1	0.031	0.8820	0.9498	1.031
PRS.14	0	0.726	**0.0131**	**0.0367**	2.067
	1	-0.072	0.7350	0.9112	0.931
PRS.10	0	0.897	**0.0120**	**0.0367**	2.452
	1	-0.124	0.6120	0.9112	0.883
PRS.11	0	0.880	**0.0114**	**0.0367**	2.411
	1	-0.093	0.6900	0.9112	0.911
PRS.74	0	0.510	**0.0466**	0.0932	1.665
	1	0.120	0.5270	0.9112	1.127

In bold, statistically significant terms (*p* < 0.05).

## 4 Concluding remarks

This paper presents a guide based on simple steps to help researchers in PRS studies. We describe these steps and present different situations and solutions through *Working Examples* and with a real data set. The situations presented in this guide do not cover all possible scenarios. For this reason, we have prioritized the most common ones. In our opinion, this is not about showing all possible options, but rather highlighting the need for a more detailed study for some (not all) PRS that appear as prioritized candidates with a possible association with the trait. In this work, we have not considered the case of having a categorical trait with more than two categories. Since it is a situation of great interest, we will give the attention it deserves in future work. Finally, this guide is focused on the analysis of the association of a PRS with a trait, and it does not delve into which methodology is the most appropriate or up-to-date for calculating the PRS. Nevertheless, it is important to recognize that each step in the process, from GWAS discovery to PRS calculation and the subsequent association analyses, introduces potential sources of error (e.g., limited GWAS power, imputational inaccuracies, or suboptimal PRS parameter choices), and the accumulation of these can influence the robustness and interpretation of the final results (16). Furthermore, it should not be forgotten that the accuracy of a PRS depends on the genetic ancestry of the group used to obtain it, and that it may present significantly lower accuracy when applied to other groups (17, 18). Finally, it does not detail the concepts or statistical techniques it encourages to use, since it only aims to indicate which steps should be followed to perform a correct analysis.

## Data Availability

The datasets presented in this study can be found in online repositories. The names of the repository/repositories and accession number(s) can be found below: https://github.com/ItziarI/SupportingMaterial-for-the-guide. All data sets, R code for the analysis, PDF files containing the analyses with the software, outputs for the *Working Examples*, and the real data sets can be found at https://github.com/ItziarI/SupportingMaterial-for-the-guide.
